# Current Applications of Models of Genetic Effects with Interactions Across the Genome

**DOI:** 10.2174/138920212799860689

**Published:** 2012-04

**Authors:** José M Álvarez-Castro

**Affiliations:** University of Santiago de Compostela, Department of Genetics, Veterinary Faculty, Avda. Carvalho Calero, ES-27002 Lugo, Galiza, Spain

**Keywords:** Epistasis, Functional models, Genetic networks, Models of genetic effects, Quantitative trait loci, Statistical models.

## Abstract

Models of genetic effects integrate the action of genes, regulatory regions and interactions among alleles across the genome. Such theoretical frameworks are critical for applied studies in at least two ways. First, discovering genetic networks with specific effects underlying traits in populations requires the development of models that implement those effects as parameters—adjusting the implementation of epistasis parameters in genetic models has for instance been a requirement for properly testing for epistasis in gene-mapping studies. Second, studying the properties and implications of models of genetic effects that involve complex genetic networks has proven to be valuable, whether those networks have been revealed for particular organisms or inferred to be of interest from theoretical works and simulations. Here I review the current state of development and recent applications of models of genetic effects. I focus on general models aiming to depict complex genotype-to-phenotype maps and on applications of them to networks of interacting loci.

## INTRODUCTION

Models of genetic effects are maps relating phenotypes to parameters with genetic meaning and biological insight. The phenotypes enter these models as genotypic values—the expected phenotype for each genotype—and the latter ones are the genetic effects. This connection between phenotypes and genetic effects enabled Fisher [1] to merge Mendel’s laws with the observations of the biometricians on the inheritance of quantitative traits. Thus, the genetic effects entail the core of the quantitative genetics theory, providing a basis for fundamental concepts and applications as breeding values, resemblance between relatives, heritability and response to selection (*e.g.* [2, 3]).

It is noteworthy, however, that traditional applications of quantitative genetics were (and may keep on being) carried out using information about individual phenotypes and relatedness among individuals alone. Genotypes entail the pivotal ingredient of models of genetic effects but they were largely out of reach of researchers for a long time. The quantitative genetics theory was founded on individual genotypes, but initially treated them from a black-box perspective while allowing researchers to compute parameters of interest from visual observations. Two such parameters are heritability and the best linear unbiased predictor (BLUP) for breeding values. Restricted maximum likelihood (REML) has allowed estimation of heritabilities and BLUP under a wide range of experimental situations (*e.g.* [2, 4]).

The advent of quantitative trait loci (QTL) analysis in the 1990s opened new fields for research. Many genetics research lines shifted focus to genes and genotypes as mapping phenotypes became available [5]. The ultimate goal of this approach is to locate the (most important) underlying genes and regulatory regions (the QTL) of a trait and to estimate the different effects of their allelic variants at the nucleotide level (the QTN) so that individual genotypes can be assessed and anticipated [2, 4]. Two decades later, both refined experimental designs and advanced statistical tools have been developed to aid this aim (see *e.g.* [6]). Similarly, progress has been and keeps on being made on models of genetic effects for both more efficiently mapping QTL, estimating their effects and analysing their role in evolution, disease impact or breeding programs.

In this communication, I review models of genetic effects with epistasis and current applications of them. I concentrate on models aiming to depict general genotype-to-phenotype maps and in applications of them to more or less particular cases. I begin by stressing the importance of implementing epistasis in genetic modelling. Next, I focus on models that are meant to analyse genetic properties at the population level, both from the perspective of traditional quantitative genetics and in the more recent context of QTL experiments. I draw special attention to the application of population-referenced genetic effects to genetic filtering. Then, I focus on genetic effects aiming to depict the genotype-to-phenotype map at the organism level. I recall why these models are necessary and review the terminology that has been used to express the duality between population- and individual-referenced genetic effects. I also develop an example to illustrate how individual-referenced genetic effects can be applied to model genetic networks entailing molecule interactions. Finally, I discuss current challenges in the development of models of genetic effects and their applications.

## GENETIC NETWORKS OF INTERACTING LOCI

The genetic architecture of a trait is the network of loci affecting that trait plus the effects of their alleles, both additive and interaction effects. The latter ones include within-locus interactions, like dominance and imprinting, and between- and among-locus interactions, which are pairwise and higher order epistasis, respectively. Sex-interaction effects may also occur and when different environments are considered, putative gene-by-environment interactions are included as well. Finally, pleiotropy should also be taken into account when considering more than one trait. It must be kept in mind that the genetic architecture of a trait cannot be detached from the population in which it is studied—which we will retake in the next section of this article. In other words, the loci affecting a trait in a population are the ones for which that population entails genetic variability affecting that trait. There could be, for instance, a gene holding great potential for functionally affecting a trait value that is fixed for one allele in a particular population. Such a gene could comprise an important component of the genetic architecture of the trait potentially, but it would show no effect on the trait in that population.

### Mapping Epistasis

Epistasis is known to also be able to conceal effects of genes—in this case, variable genes—in a population [7, 8]. Sign epistasis consists in one allele substitution increasing a phenotype under one genetic background and decreasing it under a different one, which is known to occur both in natural and experimental populations [9, 10]. This phenomenon may make the average marginal effect of allele substitutions within a locus to average out in an experimental population (with a particular set of genotype frequencies) so that it may be missed out in a standard QTL mapping study. As a consequence of this, epistatic effects between loci shall be estimated and tested when aiming to detect such loci, for which models of genetic effects with epistasis are required (*e.g.* [8]). This strategy of searching for epistasis genome-wide implies two-dimensional scans of the genome, which are associated to higher computational burdens and more severe multiple-testing problems than the conventional one-dimensional genome scans of marginal effects. Despite that, epistatic searches have in many cases mapped pairs of QTL that were dismissed in one-dimensional searches (*e.g.* [11, 12]) and examples of even higher order epistasis are known [13-15]. This exemplifies that even when the objective is to just locate QTL, geneticists profit from models of genetic effects that are more complex than the ones with only marginal effects.

Recently, epistasis has also been pointed out as an important issue to look at in genome-wide association studies (GWAS) [16-18]. A multiplicity of methods have already been developed and used for finding small groups of interacting loci (reviewed in [19-21]) and larger pathways [22]. As the availability of denser marker maps rapidly boosts, more efficient computational methods are developed that enable researchers to handle and take advantage from them in the quest for epistasis [23]. Whether loci are mapped through QTL analysis or GWAS, inspecting epistasis may aid our understanding of phenomena that are critical for both evolutionary genetics and genetic breeding—examples follow.

### Mechanisms Involving Epistasis

Midparent heterosis occurs when F_1_ and F_2_ hybrids are on average more vigorous than the midpoint of the two parental populations. Geneticists have tried to understand and exploit heterosis since long [24] and keep on meeting this challenge using nowadays latest molecular and statistical tools. Initially, dominance and overdominance were proposed as the simplest mechanisms that could underpin heterosis (*e.g.* [25, 26]). However, the genetic architecture of heterosis has recently been addressed using genetic modelling of QTL experiments and additive-by-additive epistasis has been identified as a major component of midparent heterosis. Based on this, a special parameter called augmented dominance effect of a locus has been defined from the dominance effect of that QTL and the additive-by-additive interaction effects with other QTL [27] . Melchinger and colleagues have used this parameter to improve QTL detection and dissect the genetic architecture of heterosis in plant populations (*e.g.* [28-32]). Several other research groups have also found epistasis to underpin heterosis (*e.g.* [33-36]).

Transgressive segregation (TS) is another deviation from what is expected under blending inheritance. It occurs when there are hybrid genotypic values lying outside the interval between the two parental populations. TS is frequent in both plant and animal populations [37] and it has been suggested that it occurs under additive genetic architectures due to antagonistic QTL, whose alleles are in the opposite direction to parental differences [38]. Although the presence of antagonistic QTL seems to be the rule rather than the exception, crosses exhibiting the highest levels of TS have not been found to be associated to an increased frequency of antagonistic QTL [38]. It is thus sensible to once again resort to epistasis as a likely causative genetic mechanism for phenotypic explanation and prediction. Indeed, traces have recently been found from experimental chicken populations supporting this proposal (Álvarez-Castro *et al.*, in preparation).

Hybrid incompatibilities occur when the offspring coming from the cross of two lines display reduced viability or fertility. This can also be described as TS on fitness since the offspring has lower fitness than any of the parental lines. Bateson, Dobzhansky and Muller [39-41] stressed the importance of hybrid incompatibilities for speciation and separately concurred in pointing out that epistasis must be a major factor in the evolution of hybrid incompatibilities in allopatric populations coming from the split of a common ancestral population—which is called the Bateson-Dobzhansky-Muller (BDM) model [42, 43]. Some cases of BDM incompatibilities are already studied at the molecular level [44]. We retake hybrid incompatibilities below, as well as other situations that emphasize the importance of epistasis in quantitative genetic studies.

## POPULATION-REFERENCED GENETIC EFFECTS

As initially formalized by Fisher [1, 45], genetic effects can be set up as regression coefficients and residuals that typify the properties of a trait in a particular population, with a given set of genotype frequencies. This way the genetic effects are defined from a least-square principle that makes them be statistically independent, *i.e*. orthogonal, at the population in question. Population-referenced models are thus functions of the population frequencies and they would optimally be general in terms of both the genetic effects and the population frequencies they can implicate. Kempthorne [46] and Cockerham [47] took up Fisher’s baton by implementing epistasis in orthogonal models of genetic effects with multiple alleles and departures from the Hardy-Weinberg proportions, respectively. These models, entailing additive-by-additive, additive-by-dominance and dominance-by-dominance effects, are the basis on which orthogonal decompositions of the genetic variance in epistatic systems can be derived (*e.g.* [3, 4, 48-51]).

### Additive Variance Under Drift and Selection

Assessing the behaviour of the components of the genetic variance under selection and drift has comprised a striking example of the use of those models. As a general principle for additive genetic architectures, the additive genetic variance should decrease and even eventually vanish in the face of drift [52]. It is also since long known, however, that this principle does not necessarily apply to nonadditive gene action—specifically to the presence of dominance [53]. Pioneering theoretical works have also shown that epistasis may cause an increase of additive genetic variance after a population bottleneck [54, 55], which led to a quantity of follow-up literature (*e.g.* [56-61]). There actually exists empirical evidence for the release of additive genetic variance after bottlenecks (see [62, 63]). It must also be kept in mind, though, that a recent simulation study has suggested that in the presence of purifying selection (under a mutation-selection-drift scenario) conversion of nonadditive into additive genetic variances due to bottlenecks occurs in specific situations only and that, due to inbreeding depression, this is unlikely to lead to a net increase of the adaptive potential of a population [64].

The theoretical study of the additive variance under selection has led to results similar to the aforementioned case of drift. Directional selection is initially assumed to remove genetic variation (*e.g.* [65]). However, several theoretical and simulation studies have shown that additive variance can be maintained or even temporarily increased in spite of directional selection when nonadditive gene action takes place [66-70]. As for the case of drift above, empirical work exists in accordance with these results [71-73]. The release of additive genetic variance of a trait under stabilizing selection after mutation or environmental changes can also be explained by the action of epistasis [74, 75].

### What the Epistatic Variance Tells Us

Following Fisher’s statistical theory [1, 3, 45], the decomposition of the genetic variance is performed in a hierarchical way so that as much variance as possible is accounted for by the additive effects (regression coefficients) and decreasing amounts of variance are available to be accounted for by within, between and among locus interaction effects (residuals of increasingly higher order). Consequently, the epistatic genetic variance of a trait is in general expected to be relatively small. Being able to compute the different components of the genetic variance, from the additive component to the epistatic ones (additive-by-additive, additive-by-dominance and so on) has proven to be valuable as a basis for concepts like the dissection of the resemblance between relatives (*e.g.* [3]). But it is necessary also keeping in mind that it would be misleading to try to use the decomposition of the genetic variance to infer all kinds of evolutionary properties. Indeed, the relative amount of epistatic variance of a trait is very poorly informative about the evolutionary role epistasis may be playing in a population (see *e.g.* [76]). I find the cases reviewed just above to be paradigmatic counterexamples supporting this claim—since they show that the presence of epistasis may actually mediate a boost of additive variance under several conditions.

On the other hand, multilocus random genotype-to-phenotype maps can be built by assigning genotypic values to genotypes using a random distribution. It has very recently been shown that doing so generates rates of epistatic variance that are higher than the ones often measured in empirical situations [77]. Therefore, empirical epistatic variance rates also tell us that real genotype-to-phenotype maps are not equivalent to completely random maps. Introducing order preservation as a constraint when artificially building the maps (*i.e*. precluding overdominance and preserving the order of allele substitutions across backgrounds, at least in some loci) has been found to generate more realistic rates of epistatic variance [77]. It would be interesting to inspect the rates of epistatic variance of architectures that are constrained by other reasonable biological assumptions, like modularity.

## POPULATION-REFERENCED MODELS AND QTL ANALYSIS

Beyond classical applications of the decomposition of the genetic variance to population and quantitative genetics, the advent of QTL analysis further fuelled the improvement of models of genetic effects. Orthogonal models of genetic effects have in general been found convenient for QTL analysis. I hereafter address three particular advantages of them.

### Model Selection

First, they are convenient for estimation of genetic effects, particularly when studying epistasis. Kao and Zeng [78] elaborated on Cockerham’s model [47]—built on a set of eight orthogonal scales for describing and estimating additive, dominance and epistatic effects of two interacting loci with two alleles each—for the particular case of an F_2_ population and particuarly stressed this point. Estimation of genetic effects with orthogonal models will not be affected by the presence/absence of other genetic effects in the model, which for instance makes estimates of marginal effects of particular loci to remain constant regardless of whether putative epistatic effects with other loci are considered in our model or not (see also [79, 80]). Therefore, orthogonality is an extremely convenient property for straightforward model selection in QTL mapping—neither the estimated genetic effects nor their variances are modified by adding/removing other putative effects from the model.

### Genetic Filtering

As a second advantage of orthogonal models of genetic effects, we here elaborate on why and how to use them to perform genetic filtering of genotypic values in a QTL study. After a model selection procedure in a QTL mapping experiment, a genetic architecture will optimally have been chosen entailing the putative genetic factors that underlay the expression of a trait in a population. This includes not only a set of loci but also a set of genetic effects, namely (data permitting) additive, dominance, imprinting, and epistatic effects as well as gene-sex and gene-environment interactions. It is worth noting that not all possible effects of the selected loci will be included in the resulting genetic architecture. Some loci may for instance show dominance effects whereas others may not; some epistatic loci may even show no additive effects under a certain genetic background, as pointed out above. Similarly, some epistatic effects between/among the selected loci shall be found to be significant whereas others shall not.

Focussing now on the genotypic values, they may be computed for each genotype as the mean phenotype of the individuals having that genotype. Two different problems may arise when doing so. On the one hand, the genotypic values computed this way will entail all kinds of genetic effects, including those that we may have discarded by a model selection procedure. Had for instance four loci been selected to significantly affect a trait, the genetic effects would entail up to four-order epistasis. This can be due to four order epistasis to actually occur—in which case we will most surely not be able to prove statistically—and/or just because our genotypic values are affected by sampling errors that show up, amongst other things, as all levels of genetic interaction. Therefore, researchers may often be interested in filtering out those genetic effects from our genotypic values.

On the other hand, above it has been assumed that it will be possible to compute the mean phenotype of each group of individuals with a particular genotype, that is, that in the data there are individuals with all possible genotypes. With a network of just four loci and two alleles per locus, the number of possible genotypes is 3^4^=81 and the expected number of individuals of each of the 22 possible complete homozygotes in an F_2_ population of 800 individuals is just 3.125—hence, some empty genotypic classes will most surely occur. Thus, it will often not be possible to compute all genotypic values directly from the phenotypic data. Opportunely, it will still be possible to compute them using genetic modelling from the set of genetic effects picked up by the model selection procedure.

**Fig. (1):**
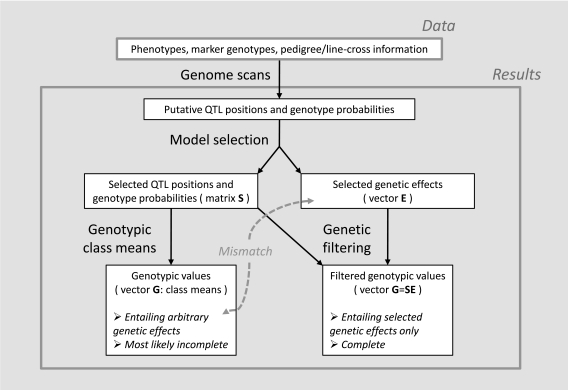
This diagram illustrates genetic filtering. After having selected a set of loci underlying a trait in a population, the genotypic values can be computed simply as means of the genotypic classes. It must be noted that this leads to genotypic values involving all genetic interactions, whether they reflect actual interactions or just arbitrary effects due to noise. In other words, there will presumably be a mismatch between the genotypic values obtained from the selected QTL positions and the selected genetic effects. Moreover, this map will often be incomplete because of lacking classes in the data. Alternatively, genetic filtering consists in computing the genotypic values using information from not only the selected positions and their genotypic probabilities (that are used to obtain the genetic-effect design matrix, S) but also from the selected set of genetic effects (the vector **E**), through the expression **G**=**SE**, so that all genetic effects discarded in the model selection procedure get to be filtered out. Using this alternative method, the resulting genotype-to-phenotype map is complete and perfectly matches the selected genetic effects. Orthogonality of the matrix S warrants that the selected genetic effects remain unchanged when removing the discarded ones.

Indeed, genetic filtering consists in using a set of genetic effects selected for a set of loci of interest to (re-)compute the genotype-to-phenotype map (see diagram in Fig. **1**). By doing so all genotypic values will be obtained entailing all genetic effects considered, and only those. However, this procedure will not make complete sense when the genetic effects that have been selected into our model change drastically after removing the ones discarded in the model selection procedure. Orthogonal models of genetic effects thus warrant genetic filtering to be performed in a consistent manner. As an example, applying genetic filtering with the natural and orthogonal interactions (NOIA) model, Le Rouzic *et al. *[81] have shown that the age at which some genes produce their effects in chicken growth are highly dependent on the genetic background.

### Interpretation

Finally, orthogonal models of genetic effects aid the interpretation of estimates obtained in QTL analyses. Orthogonal additive effects are average effects of allele substitutions in populations [1, 2]. These are the basis for the definition of breeding values and they enable performing a decomposition of the genetic variance with no genetic covariances, which in its turn sets the basis for the mathematical description of heritability (*e.g.* [2]). Thus, estimates of genetic effects from a QTL study have a clear interpretation as they are descriptors of the genetic properties of the mapping population [78-80].

Further, note that mapping populations are often ad-hoc experimental constructs that have been built with the only purpose of identifying a set of loci underlying a trait. Researchers will thus often be interested in additional information about the genetic properties of populations different from the mapping population. Conveniently, estimates of genetic effects associated to a population can be straightforwardly transformed into the genetic effects of another population (with a different set of genotype frequencies) as long as orthogonal models of genetic effects have been described for both populations [82]. Overall, orthogonal models of genetic effects aid mapping and interpretation of the genetic architecture detected in a QTL study beyond the framework of the particular population used for mapping the QTL.

## INDIVIDUAL-REFERENCED GENETIC EFFECTS

We have just discussed why models of genetic effects based on average effects of allele substitutions over populations are the core of quantitative genetics theory and we have in particular mentioned that, in QTL analyses, obtaining genotypic values that are coherent with a particular genetic architecture is one of the advantages we can gain from those models. It is at this point worth noting that genotypic values are however assumingly independent of the genotypic frequencies of the population in which they are measured. Consequently, it could be considered that providing genetic effects also in a population-independent way could enable researchers to attain further interpretation. Actually, Fisher [1] already proffered a simple non-population-referenced model, using sort of arbitrary additive and dominance effects as an initial yardstick on which to root further statistical developments. Somehow paradoxically, those arbitrary effects were later called the F_∞_ model because they can also be interpreted population-wise—they are orthogonal at an ideal population resulting after infinite generations of brother-sister mating [80, 82, 83]. From the point of view of a QTL experiment, this F_∞_ population does not allow estimation of dominance effects—nor any kind of epistatic effects involving dominance, like additive-by-dominance effects.

In any case, the early attempts to map QTL [84] already gave rise to the need of models of genetic effects that were not focused on population gene or genotype frequencies. Indeed, Jana [85] found the non-population-referenced model by Seyffert [86] to be more adequate than population-referenced ones in aiding “physiological and biochemical interpretation of dominance and epistasis”. In the more recent context of interval mapping [5, 87, 88], the same concern has been put forward by Cheverud and Routman [89, 90]. They developed their physiological model aiming to depict functional interactions between molecules resulting from the expression of loci at the organism level, rather than the averages of the effects of those loci within particular populations as depicted in the statistical models. As well as Fisher’s [1] average (statistical) genetic effects, Cheverud and Routman’s [90] can be obtained as regression coefficients and residuals, the difference being that the first ones result from a regression weighted by the genotypic frequencies of a population whereas the later ones result from an unweighted regression—thus being population-independent.

### Multilinear Epistasis

Hansen and Wagner [91] further developed Cheverud and Routman’s [90] ideas into an individual-referenced model framework. Their multilinear model implements epistasis using a multilinear framework in which functional genetic effects are given a biological, population-independent meaning as effects of allele substitutions from reference individual genotypes. Further, Hansen and Wagner [91] introduced the concept of change of reference of genetic effects and developed expressions to transform the (multilinear) genetic effects from the reference of an individual genotype into the ones from a different reference genotype. The multilinear model can also be linked to population-referenced genetic effects through constructing hypothetical individuals whose alleles have effects that are averages of the effects of the alleles present in a population.

The multilinear model implements epistasis in an analytical tractable manner and it has proven to be useful for analyzing the potential evolutionary properties of the genetic architecture of a trait. In particular, Hansen and colleagues [69, 92] have found directionality to be a key property in determining the outcome of a population’s response to directional selection pressures along many generations. A parameter has been described to measure directionality, the directional epistasis coefficient [69]. This parameter can be—and has actually already been—used to obtain empirical estimates of directionality [93, 94]. Directionality is thus, similar to heritability, a predictor of the kind of response to selection of a trait in a population. Further, it is noteworthy that directionality conditions response to selection in a longer timeframe than heritability does.

The multilinear model has also been applied to studies involving stabilizing selection. The expected equilibrium genetic variance under mutation-selection-drift balance has been found to decrease with multilinear epistasis [95, 96]. On the other hand, Fierst and Hansen [97] have pointed out that opposed directional selection is not a necessary condition at all for hybrid incompatibilities to evolve under the BDM model. To the contrary, they may occur between isolated populations under stabilizing selection—even when sharing the same optimum—with weak or moderate multilinear epistasis.

### A General Functional Formulation

Functional genetic effects can also be modelled as natural effects of allele substitutions from individual genotypes in a theoretical framework with completely general—*e.g.* not necessarily multilinear—epistasis. The traditional, statistical models cannot be accommodated to do this because their reference points are intercepts of regressions weighted by the population frequencies—hence, setting the population frequencies to use one individual genotype as intercept is equivalent to trying to perform a regression to a single point. Thus, a new set of formulae had to be developed for this task. For the biallelic case, Álvarez-Castro and Carlborg [82] modelled the natural effects of allele substitutions of the functional models in an analogous way to the orthogonal, averaged effects of allele substitutions of the statistical models. Using a consistent notation enabled Álvarez-Castro and Carlborg [82] to make functional and statistical models of genetic effects to be straightforwardly interchangeable—in a way similar to how Van der Ween [98] connected a few genetic models that were used at the time. Functional and statistical genetic effects are this way unified into NOIA, a model integrating both natural (single substitution effects) and orthogonal parameters. Yang and Álvarez-Castro [99] extended the functional formulation of the NOIA model to arbitrary numbers of alleles at each locus. Table **1** shows a summary of the properties of several models of genetic effects.

Free software (noia [93]) has been released for the application of NOIA to empirical data. Estimates of effects of allele substitutions from an ancestral chicken genotype to a domesticated one have been obtained using this software [100]. Several other publications have applied noia to study epistasis in the domestication and selection response of chicken [13, 81, 101, 102] and also to understanding the genetics underlying tameness in rats [101, 103]. Also using the noia software, Pavlicev *et al. *[94] have obtained estimates of coefficients of directional epistasis in mice.

## NOMENCLATURE

One of the premises that enormously smoothes the progress of a field is to establish a common, unambiguous terminology. Above, we have on the one hand labelled the population-referenced models of genetic effects as statistical models, which describe the genotype-to-phenotype map orthogonally as a set of averaged allele substitutions from the mean of a population. On the other hand, we have labelled as functional models the ones describing the genotype-to-phenotype maps as sets of natural allele substitutions from the reference of individual genotypes. Indeed, epistasis raised a debate concerning the different approaches to formulating genetic effects (*e.g.* [80, 82, 90, 91]) and it is interesting to note that the duality between functional and statistical parameters also concerns models with only marginal genetic effects [82, 99].

Both the above mentioned physiological [90] and multilinear [91] models focus on the phenotypic effects of alleles at the organism level in a non-population-referenced way. This angle has been referred to as functional formulations of genetic effects or functional epistasis (*e.g.* [10, 82, 91])—especially when using individual genotypes as reference points—but it has also been referred to as biological [104] and even split into genetical and biological, for distinguishing interactions between DNA sequences and between proteins, respectively [105]. More recently, also the label compositional has been proposed to track effects of allele substitutions from reference individual genotypes while suggesting to keep the label functional for more explicitly referring to how molecules function and interact [106]. Indeed, functional epistasis was used to refer to phenotype and fitness effects of physiological facts since long (*e.g.* [107]) and keeps on being used in that sense at present [108-111].

The duality between formulations of genetic effects has also been expressed as Cockerham type versus F_∞_ type models [112]. Although not completely clearly defined, this divide seems to match the one mentioned above—statistical versus functional (or compositional) formulations. The choice of Cockerham to name the first type of models seems somehow arbitrary since Cockerham [47] developments are on the one hand extensions of Fisher’s [1] and on the other hand less general than Kempthorne’s [3, 46] (see *e.g.* [113]). More importantly, the F_∞_ model has been regarded both as a sort of arbitrary, population-independent model and as a population-dependent one, as explained above, which makes it confusing to use it as a label for one of these two types.

To sum up, a huge gap seems to be perceived between some self-proclaimed functional formulations of genetic effects and the modelling of the action of molecules at the physiological level (see *e.g.* [16, 106, 114, 115]). When initially proposing the label functional for individual-referenced models, Hansen and Wagner [91] deemed effects of allele substitutions from reference individuals to certainly be more informative about how gene products function than average effects over populations could be. To further clarify this point, I devote the next section of this communication to develop an example illustrating how the flexibility of those functional formulations aids the modelling of specific molecule interactions of evolutionary significance, leading in particular to hybrid incompatibilities. Currently lacking a general consensus, throughout this communication I keep on using the label functional *sensu* Hansen and Wagner [91].

## MODELLING MOLECULE INTERACTIONS WITH A FUNCTIONAL FORMULATION

Let us here consider a case of hybrid incompatibilities in accordance with the BDM model, with two loci, A and B, which code for two different protein monomers that bind into a functional enzyme dimmer. It will be assumed that one population is initially fixed for alleles “1” at both loci, A_1_ and B_1_. Next, that population splits into two isolated populations where new alleles at the two loci, A_2_ and B_2_, arise by mutation and get eventually fixed—one allele in each population. Then, the two isolated populations merge and the “2” alleles of loci A and B, which were compatible with the “1” alleles, happen not to be completely compatible with each other. Two possible types of molecule interaction are considered that can underpin this lack of compatibility.

First, let us consider the possibility that alleles A_2_ and B_2_ lead to enzyme monomers that bind easily but lead to non-functional enzyme dimmers—case C1. In this case, the concurrence of alleles A_2_ and B_2_ in one individual leads to a disadvantage due to a waste of resources in the production of non-functional enzyme dimmers. Second, consider that the monomers produced by alleles A_2_ and B_2_ bind difficultly—case C2. In this case, a disadvantage arises whenever an allele “2” is present at one locus only—either A_2_ or B_2_ but not both of them together—due to a waste of resources in the production of a certain amount of useless monomers.

**Fig. (2):**
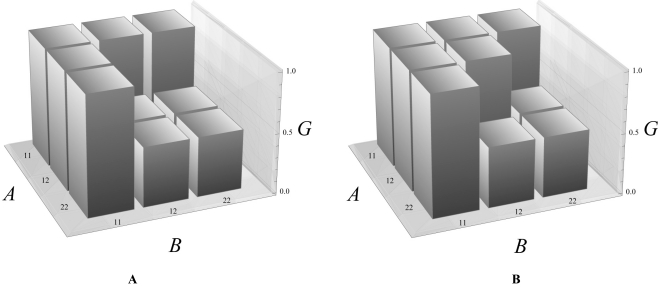
Two-locus (A and B) two-allele (1 and 2) genotype-to-phenotype maps that are consistent with the cases of hybrid incompatibility considered in the text. The genotypic values (G) consistent with cases C1 and C2 are in charts 2A and 2B, respectively. The only difference between the two cases affects the double heterozygote, which is penalized by hybrid incompatibilities in case C1, but not in case C2.

These two cases are illustrated in Fig. (**2**). Both charts **2A** and **2B** show that, in accordance with the assumptions, the populations in which the mutations appear and eventually get fixed (leading to genotypes A_1_A_1_B_2_B_2_, A_2_A_2_B_1_B_1_) keep the same trait value as the ancestral population (A_1_A_1_B_1_B_1_). By chosing that trait value to be one, it can be regarded as a relative fitness value. In Figs. (**2A** and **2B**) it can also be observed that some of the genotypes resulting from the hybridization of the two populations with fixed mutations get penalized phenotypes (particularly, to a value of ½). The only difference between the two cases is the double heterozygote, which is penalized when considering the first case of molecule interaction, C1 (Fig. **2A**), but not when considering the second one, C2 (Fig. **2B**), in accordance with our assumptions. It is thus obvious that the two cases may have different evolutionary outcomes, since an F_1_ population would be penalized in one of the cases but not in the other one.

Sets of genetic effects that reflect the two extreme cases considered, C1 and C2, and also all intermediate cases, are shown in Table **2**. The first two rows of this table gather the genetic effects for the two extreme cases as natural effects of allele substitutions from the reference of the ancestral population using the functional formulation of the NOIA model [82]. In the third row of Table **2**, the two cases are generalized by adding two parameters. The first parameter, *e*_1_, stands for the strength of the penalization. It ranges from zero (no penalization) to one (the phenotype of the penalized individuals drops to zero). Thus, this parameter takes a value of ½ in the examples of Fig. (**2**). Using the second parameter, *e*_2_, it is possible to consider the two extreme cases, C1 and C2 (for values zero and one, respectively), plus all intermediate instances. Let us now consider a population at equilibrium in which the frequencies of alleles coming from the population where B_2_ got fixed are half of the frequencies of the alleles coming from the population where A_2_ got fixed (Table **3**). The statistical genetic effects for that population can easily be obtained using the transformation tool of NOIA [82] and they are shown in the fourth row of Table **2**.

I have developed this example in order to stress three points. First, note that the functional formulation from the reference of the ancestral genotype leads to simpler expressions for the genetic effects than the statistical formulation does (rows 3 and 4 in Table **2**). I have used a functional formulation from an appropriate reference because this can be extremely helpful in modelling genetic architectures in general, and situations expressed in terms of molecule interactions in particular. Second, in spite of having used a particular (and convenient) reference point to formulate our model, we are not restricted to that choice. To the contrary, having obtained a particular formulation of genetic effects from a particular reference point, the transformation tool of the NOIA model allows easy transformation of these genetic effects into the ones fitting to a different formulation that could be convenient for the analysis—in this example, the statistical formulation at the mean of the population shown in Table **3**. Finally, the expressions of the genetic effects obtained in this example could now be used to test whether genetic effects of pairs of loci detected through QTL analyses or GWAS could fit to the kind of interactions that I have considered in this model.

## PERSPECTIVE

Significant progress in the understanding of the evolution of genetic architectures has been and will keep on being made by developing *ad hoc* models fitting specific situations of interest. For instance, the evolution of epistasis under selection has been analyzed using loci that act as modifiers of the marginal effects of other loci or of the interaction effects between other loci [116-121]. In this communication, I have chosen to focus instead on models of genetic effects aiming to represent a wide range of genetic architectures and that can then be applied to analyze numerous particular instances.

### Unifying Prospects

Several models that were initially proposed for restricted situations have later on been generalized in different ways. The F_2_ model is the most renowned case of Fisher’s [1, 45] population-referenced formulations of genetic effects. Its name reflects that it is orthogonal at (and thus it entails the genetic properties of) an ideal F_2_ population, with allele frequencies of ½. Fisher’s theory of genetic effects already was more general than that, actually parameterized to account for any biallelic frequencies so that it could be applied to different populations under the Hardy-Weinberg proportions. As mentioned above, Cockerham [47] introduced genotype frequencies in his two-locus models with epistasis—thus extending their use to nonequilibrium populations—while Kempthorne [3, 46] developed epistatic models with multiple alleles. All these approaches have recently been generalized under the framework of the NOIA model [48] (see Table **1**).

Also Fisher’s [1] initial proposal of a non-population-referenced yardstick for genetic effects—the F_∞_ model (see [83])—eventually evolved into parameterized formulations. In this case, the parameters take different values in order to represent genetic effects of multiple multiallelic loci as effects of allele substitutions from individual genotypes [91]. This approach has also been generalized to account for arbitrary dominance and epistatic interactions under the framework of the NOIA model [82, 93, 99]. NOIA has not only been intended for generalizing previous statistical and functional models of genetic effects with epistasis, but also for unifying them under a common framework, thus enabling researchers to transform genetic effects between different meanings (see Table **2** and related text for an example). The different formulations of genetic effects are required to be interchangeable because different ways of measuring genetic effects are required for addressing different particular issues and many issues are always involved in the resolution of a broad research query [106].

### Current Theoretical Challenges

Extending such a unifying framework to further genetic architectures and population facts remains a challenge. Concerning genetic effects, Wolf *et al. *[122] have recently pointed out imprinting as a significant component of genetic architecture in mice. In this work, a fixed (*i.e*. not parameterized by *e.g.* population frequencies) set of indexes was chosen to develop a model to test for imprinting. That such a model could also have been built from Cheverud and Routman’s [90] physiological model, automatically entailing an extension to physiological epistatic interactions involving imprinted loci. In any case, parameterized expressions for imprinting effects—both statistical and functional and including the one by Wolf *et al.* [122] as a particular case—would aid detection and interpretation of imprinting effects in QTL analyses. There also is a lack, to the best of my knowledge, of appropriate parameterized models for gene-by-environment interactions.

Concerning now population facts, several works have addressed the implementation of linkage disequilibrium (LD) in models of genetic effects. Cockerham [47] already mentioned the difficulty of developing models simultaneously accounting for LD and epistasis. More recently, Yang [79] derived a parameter to measure the deviation from orthogonality caused by the presence of LD in models of genetic effects with epistasis. Wang and Zeng [112, 113] studied the same issue through the covariances that occur in the decomposition of the genetic variance due to LD. Mao *et al. *[123] extended Kempthorne’s [46] epistatic model to estimate genetic effects independently in populations with departures from the Hardy-Weinberg proportions and LD. Similar to the previous attempts, their model is however not orghogonal in the presence of LD, which remains a challenge in genetic modelling.

Overall, considering epistasis largely increases the conceptual and mathematical complexity of models of genetic effects. However, this turns out to be an inescapable endeavour because of the combination of two facts. First, as pointed out throughout this communication, the effects of epistasis on response to selection and other agents are in general far from negligible. Second, evidence has been gathered that points to epistasis as a ubiquitous phenomenon in natural and experimental populations (also pointed out through some examples in this communication and extensively reviewed in the last few years [14, 17, 89, 105, 106, 124-131]).

### Dissecting Genetic Networks

Products of genes from all over the genome are known to interact through regulatory processes and physiological pathways in the assembly of traits. In the light of this biological reality, it would signify an enormous surprise that genes underlying traits showed in general scarce interaction effects. From this angle, epistasis may be considered as a null hypothesis—lack of interaction must be proven rather than assumed—whenever aiming to dissect the genetic architecture of any particular trait. At which specific point has an analysis reached enough power to demonstrate that significant epistatic interactions have not been found because they actually do not exist remains, however, not completely clear. The same kind of problem arises for being able to assess the true pattern of epistatic interactions among loci (even when having evidences for the presence of some kind of epistasis) and even to detect marginal effects with relatively small contributions to the trait variance. The arrival of routine QTL analysis about two decades ago might have made some researchers believe that mechanically disentangling biological systems would be just around the corner and, particularly, that genetic applications to human disease and to livestock production would spring out naturally (see *e.g.* [132]). But in time, several difficulties of using genome scans for portraying phenotypes at the molecular level were pointed out (*e.g.* [133]). Currently, effort is being made to keep on stepping forward in dissecting genetic architectures of traits—and further developing models of genetic effects provides us with one of the pieces required to solve that puzzle.

In the meantime, from the pioneering developments by Meuwissen *et al. *[134], genomic selection has emerged as a novel way of harnessing the rapidly growing information available on molecular markers across the genome, particularly for alleviating costly phenotyping of sex-limited traits. Genomic selection could be regarded as applying the force of vastly dense marker panels over enhanced statistical machinery for catapulting the black-box perspective of quantitative genetics towards improved genetic breeding of large commercial populations. It is noteworthy that this involves in principle no way of dissecting genetic networks since that black box contains the very practical, although biologically unrealistic, Fisher’s infinitesimal model—a large number of additive loci with small effects [135]. It is however remarkable that some contributions with more complex models of genetic effects have already been applied to genomic selection—Toro and Varona [136] have implemented dominance and several other authors have considered epistatic interactions using the NOIA model [137, 138]—supposedly at the cost of increasing data demands. It is for sure that, as QTL analysis and GWAS did before, genomic selection opportunely constitutes a breath of fresh air for the molecular-biology industry. Other than that, the quantitative genetics community has after almost a century of experience learnt to embrace with cautious optimism (*sensu* Dekkers [139]) the expectations of commercial and scientific applications coming from the successive innovative tools that are from time to time proposed to enrich this field.

## Figures and Tables

**Table 1.** Summary of properties of models of genetic effects. All models shown have parameterized statistical formulations although not all have been mathematically expressed in matrix notation or in terms of allele substitutions from individual genotypes (functional formulations). Alternatively, different models describe different genetic architectures (epistasis types, numbers of loci or alleles) and provide orthogonal decompositions of the genetic variances under different population facts (HWD or LD). Actually no model is orthogonal under LD but there are two models assessing the impact of LD on orthogonality

	Multilinear^1^	Yang’s^2^	G2A^3^	Wang and Zeng’s^4^	NOIA^5^
Matrix notation	No	Yes*	Yes	No	Yes
Functional	Yes	No	No	No	Yes
Epistasis	Multilinear	Yes	Yes	Yes	Yes
Multiple loci	Yes	Two	Yes	Two	Yes
Multiple alleles	Yes	No	No	Yes	Yes
HWD	No	Yes	No	Yes*	Yes
LD	No	Assesses	No	Assesses	No

1 By Hansen and Wagner [91].

2 Yang’s model [79] is based on Cockerham’s setting [47].

3 By Zeng et al. [80].

4 Wang and Zeng’s model [112, 113] is based on Kempthorne’s setting [46].

5 By Álvarez-Castro, Carlborg and Yang [48, 82, 99].

* Yang’s model notation is based on matrices, although different from the G=SE formulation. Wang and Zeng’s model initially considers HWD although not for the main results attained.

**Table 2.** Genetic effects of hybrid incompatibilities due to molecule interactions of gene-products of loci A and B. The first three rows are functional formulations from the reference of A_1_A_1_B_1_B_1_, the third one being a generalization of the particular cases shown in the other two. The fourth row is a statistical formulation from the reference of the population shown in Table 3 that also generalizes the two cases considered (see text for details). To be precise, the names of the parameters for the statistical case (fourth row) should actually be Greek letters (*µ* instead of R, *α* instead of *α* and *δ* instead of *d*)

	*R*	*a*_A_	*d*_A_	*a*_B_	*d*_B_	*aa*	*ad*	*da*	*dd*
Case C1	1	0	0	0	0	‑1/8	‑1/8	‑1/8	‑1/8
Case C2	1	0	0	0	0	‑1/8	‑1/8	‑1/8	3/8
Functional	1	0	0	0	0	‑*e*_1_/4	‑*e*_1_/4	‑*e*_1_/4	*e*_1_(*e*_2_‑1/4)
Statistical	1+(2^3^/3^4^) *e*_1_(2*e*_2_‑5)	(1/3^3^) *e*_1_(2^2^*e*_2_+5)	(1/18) *e*_1_(2^3^*e*_2_‑5)	(2^4^/3^3^) *e*_1_(*e*_2_‑2^2^)	‑(1/3^2^) *e*_1_(*e*_2_+2)	(1/3) *e*_1_(*e*_2_‑1)	(2^2^/3^3^)*e*_1_(*e*_2_‑1)	‑(1/6)*e*_1_(2*e*_2_+1)	*e*_1_(*e*_2_‑1/4)

**Table 3.** Genotype frequencies of an equilibrium population with double the amount of alleles A_2_ and B_1_ than A_1_ and B_2_ (see text for details)

	A_1_A_1_	A_1_A_2_	A_2_A_2_
**B_1_B_1_**	2^2^/3^4^	2^4^/3^4^	2^4^/3^4^
**B_1_B_2_**	2^2^/3^4^	2^4^/3^4^	2^4^/3^4^
**B_2_B_2_**	1/3^4^	2^2^/3^4^	2^2^/3^4^

## ABBREVIATIONS

BDM: = Bateson-Dobzhansky-Muller
BLUP: = Best unbiased linear predictor
GWAS: = Genome-wide association study
NOIA: = Natural and orthogonal interactions
QTL: = Quantitative trait loci
QTN: = Quantitative trait nucleotide
REML: = Restricted maximum likelihood
TS: = Transgressive segregation
